# Case Report: Prenatal Diagnosis of Nemaline Myopathy

**DOI:** 10.3389/fped.2022.937668

**Published:** 2022-07-19

**Authors:** Dongmei Liu, Jiali Yu, Xin Wang, Yang Yang, Li Yu, Shi Zeng, Ming Zhang, Ganqiong Xu

**Affiliations:** ^1^Department of Ultrasound Diagnostic, The Second Xiangya Hospital, Central South University, Changsha, China; ^2^Research Center of Ultrasound Diagnostic, The Second Xiangya Hospital, Central South University, Changsha, China; ^3^Clinical Research Center for Medical Imaging in Hunan Province, Changsha, China; ^4^Department of Obstetrics and Gynecology Prenatal Diagnosis Center, The Second Xiangya Hospital, Central South University, Changsha, China

**Keywords:** nemaline myopathy, arthrogryposis multiplex congenita, *KLHL40* gene, amyoplasia, prenatal diagnosis

## Abstract

Nemaline myopathy (NM) is a rare, hereditary heterogeneous myopathy. Fetal NM has a more severe disease course and a poorer prognosis and is usually lethal during the first few months of life. Hence, early prenatal diagnosis is especially important for clinical interventions and patient counseling. We report the case of a fetus with NM due to *KLHL40* gene variation leading to arthrogryposis multiplex congenita (AMC). The ultrasonography and histopathology results revealed an enhanced echo intensity and decreased muscle thickness, which may be novel features providing early clues for the prenatal diagnosis of NM. Moreover, to our knowledge, this article is the first report to describe a case of NM associated with complex congenital heart disease (CHD).

## Introduction

Nemaline myopathy (NM) is a rare congenital disease of skeletal muscle causing severe muscle weakness and other neuromuscular dysfunction manifestations. Shy first described it in 1963 (1) and Kaspersky first reported it as a prenatal diagnosis in 2008 (2). The incidence of NM is approximately 1:50,000 (3). Based on the time of onset and severity of symptoms, it can be classified into six forms ranging from severe congenital/neonatal onset to adult onset (4). The severe congenital subgroup accounts for 15% of all NM cases (5). NM is generally considered a hereditary disease, but its inheritance is heterogeneous, either dominant or recessive, and many loci are involved. Thus, NM causes various diseases. Variations in at least 12 genes including *ACTA1, CFL2, KBTBD13, KLHL40, KLHL41, LMOD3, MYPN, NEB, TNNT1, TNNT3, TPM2*, and *TPM3*, have been identified to be associated with the condition (6). Variations in the *KLHL40* gene have been recently identified as a cause of severe autosomal-recessive NM. The diagnosis of NM is mainly based on genetic and pathological features. The characteristic pathological change is the deposition of a large number of nemaline bodies in muscle fibers. Prenatal ultrasound diagnosis of NM is difficult and thus rarely performed. This study aimed to describe novel ultrasound findings for the lethal form of NM. Enhanced echo intensity and decreased muscle thickness demonstrate that amyoplasia seems to be an element of the poor prognosis of these patients. Thus, when ultrasonographic features such as polyhydramnios, decreased fetal movements and club feet are noted during pregnancy, the echo and thickness of the muscle should be observed carefully to determine the potential existence of congenital neuromuscular disorders such as NM.

## Case Report

A 36-year-old Chinese woman, gravida 1, para 0, was referred to our hospital at 36 weeks of gestation because of multiple cardiac anomalies (right ventricular double outlet and aortic coarctation) and bilateral foot inversion observed in the fetus. At 18 weeks gestation, amniocentesis showed that the fetus was at high risk for trisomy 18 and trisomy 21. The chromosomal microarray of amniocytes was normal. No exposure to teratogenic substances or family history of congenital malformation was noted. The pregnancy was uncomplicated.

**FIGURE 1:**
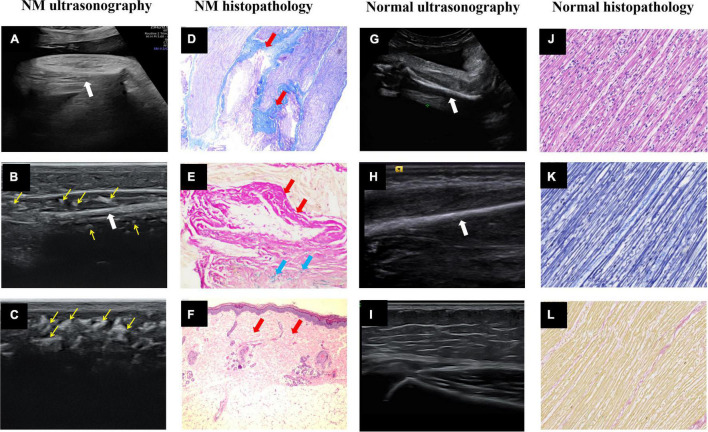
Ultrasound images and histopathology of the prenatal and postnatal extremities. **(A)** Intrauterine ultrasound examination. **(B,C)** Postpartum high-frequency probe examination showed NM fetal upper limb muscle echo close to the humerus echo, and many hyperechoic regions (thin yellow arrows) in the muscle layer and subcutaneous tissue. **(D)** Right calf muscle Masson trichrome staining × 40 and **(E)** right calf muscle elastic fiber staining × 200. The muscle fiber fascicles were sparse and replaced by collagen fibers (thick red arrows) and elastic fibers (thick blue arrows). **(F)** Right calf skin H&E × 40 showed fibrous tissue (thick red arrows) proliferated in the dermis layer and subcutaneous tissue. **(G–I)** The normal fetal upper limb muscle echo was significantly lower than the humerus echo, and the subcutaneous tissue had low echo intensity with several echogenic septa of connective tissue. The thick white arrows point to the long bones of the fetal limbs. **(J–L)** The normal fetal right calf muscle H&E × 40, Masson trichrome staining × 40 and elastic fiber staining ×40.

**FIGURE 2:**
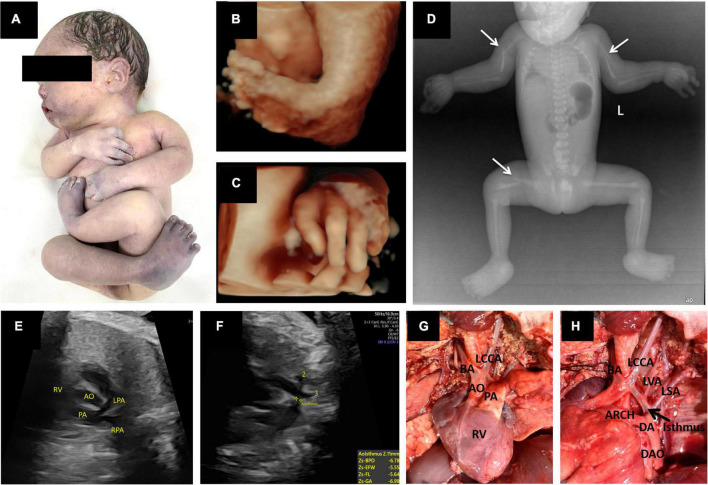
Prenatal 3D ultrasound images and fetal echocardiography, postpartum gross anatomical specimens, X-ray photographs and cardiac anatomy. **(A)** Postmortem photo demonstrated multiple contractures of the upper and lower extremities. **(B,C)** Three-dimensional ultrasound showed fetal foot inversion and tilted fingers. **(D)** X-rays showed bilateral humerus and right femur fractures (thin white arrow). **(E,F)** Prenatal echocardiography showed a right ventricular double outlet with coarctation of the aortic arch. **(G,H)** Postpartum cardiac anatomy confirmed sonographic findings. In addition, there were four arteries of the aortic arch, from right to left: BA, the brachiocephalic artery; LCCA, the left common carotid artery; LVA, the left vertebral artery; and LSA, the left subclavian artery. RV, right ventricle; AO, aorta; PA, pulmonary artery; LPA, left pulmonary artery; RPA, right pulmonary artery; DA, ductus arteriosus; DAO, descending aorta; ARCH, aortic arch.

The first ultrasound examination conducted in our department showed polyhydramnios, and the fetus was in breech presentation with no obvious limb movements, bilateral clubfoot, or tilted fingers. The cardiac malformation was the same as before. Interestingly, we found that echoes of the skeletal muscles were significantly enhanced (close to or slightly weaker than the bone echo) and had posterior echo attenuation (Figures 1A,B). Serial scans were performed the following week, which confirmed limb stiffness (Figures 2B,C). In addition, it was found by comparison that limb muscles of this fetus were thinner than those in the control. Ultrasonographic findings led to the suspicion of AMC associated with amyoplasia, complex congenital heart disease (CHD) (Figures 2E,F), and polyhydramnios.

Whole-exome sequencing (WES) was then performed on fetal umbilical cord blood and revealed a homozygous variation in the *KLHL40* gene, NM_152393.2:c.1516A > C (p. Thr506Pro). Sanger sequencing verified that the patient was homozygous variation for c.1516A > C (p. Thr506Pro) (Supplementary Figure 1). According to ACMG guidelines, the variant was classified as pathogenic (PM1 + PM2 + PM3 + PP1 + PP4).

**FIGURE 3:**
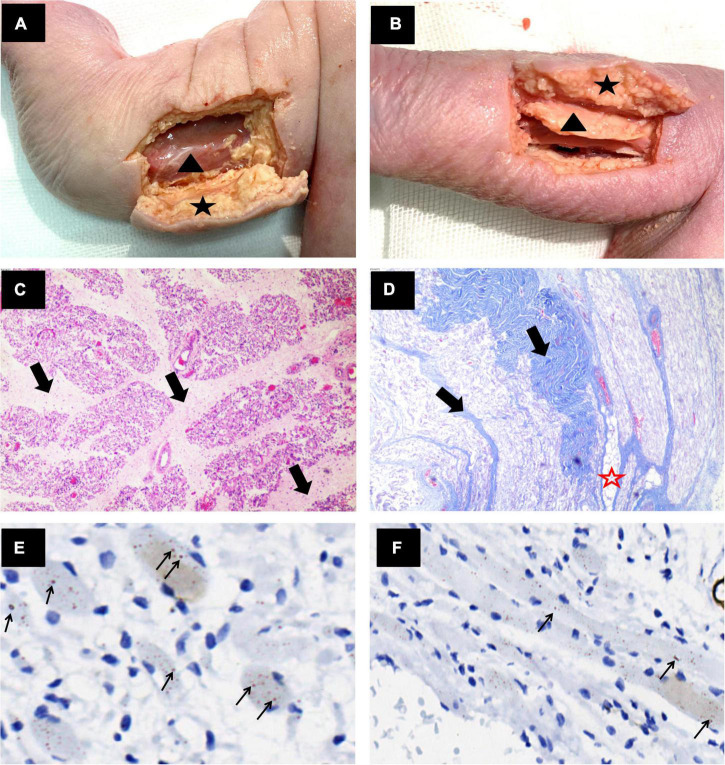
Anatomical and pathological images of the limb muscles. **(A,B)** Gross anatomy showed thickened subcutaneous fat (black star) and a thin muscle layer (black triangle) in the lower limbs of the fetus. **(C)** Right thigh muscle cross section H&E × 40 and **(D)** Right forearm muscle longitudinal section Masson trichrome × 40. The muscle bundles showed marked variation in size, and muscle fibers were replaced by fibrous tissue (thick black arrows) and adipose tissue (red star). **(E,F)** Right calf muscle cross section and longitudinal section anti-α-actinin antibody stained × 400. The muscle fibers were sparse, degenerated, and atrophied. Lots of nemaline rods in muscle fibers were strongly stained with anti-α-actinin antibody (thin black arrow).

The pregnancy was selectively terminated after the patient received counseling. Postmortem radiological examination, necropsy (Figures 2A,D), and pathology confirmed all sonographic findings and revealed other malformations. X-rays showed pathological fractures. The autopsy revealed that the skin had increased hardness and decreased elasticity. The subcutaneous fat layer was significantly thickened, and the muscle layer was significantly thinner (Figures 3A,B). In addition, there were four arteries in the aortic arch (Figures 2G,H).

Histopathological examination showed the following: (1) The muscle fibers were sparse, degenerated, and atrophied and were replaced by fatty-fibers (Figures 3C–F). (2) The dermis layer and subcutaneous tissue were thickened with fibrous tissue proliferation (Figure 1F); and (3) Nemaline bodies were positively stained with the anti-α-actinin antibody (Figures 3E,F). Unfortunately, we failed to perform electron microscopy. Altogether, the results of these examinations indicated a diagnosis of nemaline myopathy due to *KLHL40* gene variation leading to fetal AMC associated with CHD.

## Discussion

AMC is a heterogeneous disease characterized by multiple congenital joint contractures. This is the result of reduced fetal movement during development. AMC has been shown to be associated with more than 400 diseases and 350 known genes. AMC has been reported in association with neuromuscular disorders. NM is one of the congenital myopathies that leads to AMC (7). Fetal NM is a severe form of NM that carries a poor prognosis. Fetal NM due to variated *KLHL40* is one of the most severe subgroups characterized by fetal akinesia (8), contractures, severe muscle weakness, respiratory failure, dysphagia, and early perinatal death (average age of death is 5 months) (9, 10). Currently, there is no available cure for fetal NM, and it has an extremely poor prognosis. Thus, early prenatal diagnosis is especially important. Several studies have suggested that prenatal identification of NM is challenging and can only be achieved by WES or fetal muscle biopsy. However, less than half of the muscle histology results of suspected NM cases present with nemaline bodies (5, 11), and WES cannot be performed for all fetuses. Therefore, prenatal ultrasound is one of the best indicators of the need to perform further WES analyses of affected fetuses.

To date, 34 prenatally diagnosed cases of NM have been reported in the literature (Table 1). Decreased fetal movement (74%), polyhydramnios (65%), and arthrogryposis (47%) were common prenatal features on sonography. Other less frequent findings included fetal edema (32%), clubfoot (26%), facial abnormalities (15%), lung hypoplasia (6%), and fractures (3%). Notably, our study is the first to describe the features of enhanced echo intensity and decreased thickness of skeletal muscle. In normal fetuses, muscle boundaries are clearly visible and the skeletal muscle echo is lower than or close to the liver echo. However, this fetus appeared to have unclear muscle boundaries. The muscle layer appeared thinned and had an enhanced echo (stronger than the liver echo and slightly weaker than or even approaching the bone echo, Figures 1A,G). Then, we used a high-frequency probe to compare the skin and muscle of this fetus to that of a normal infant (Figures 1B,C,H,I). Strikingly, the fetus demonstrated replacement of myofibers with connective tissue on ultrasonography (many hyperechoic regions in the muscles) and histopathology (Figures 1D,E). These were in stark contrast to the muscles of normal fetus (Figures 1J–L). Moreover, fibrous tissue proliferated in the dermis layer and subcutaneous tissue (Figure 1F), which exhibited several hyperechoic regions, replacing the several echogenic septa observed on ultrasonography (Figures 1C,I). Increased interstitial connective tissue is indicative of myopathy (12). In this case, the echo intensity increased because the muscular architecture was disrupted by muscle cell replacement with connective and fat tissue. These antenatal signs demonstrated that amyoplasia seemed to contribute to the poor prognosis. We recommend that decreased fetal movements combined with polyhydramnios should be used to observe the fetal joint activity and the echo and thickness of the skeletal muscle. Improving awareness and identification of abnormal ultrasound manifestations early alerts clinicians of the potential existence of neuromuscular disorders such as NM.

**TABLE 1:** Summary of prenatal ultrasound characteristics of nemaline myopathy.

Case	References	Gender	GA (weeks)	DFM	Poly	Arth	Fetal edema	Clubfoot	FA	LH	Fractures	MEE	MTT	Follow up
1	Vuopala et al. (13)	Male	INA	NS	NS	+	+	NS	NS	+	NS	NA	NA	Death (4 h)
2a	Vendittelli et al. (18)	Male	33	+	+	NS	NS	NS	NS	NS	NS	NA	NA	Death (6 days)
2b		Female	INA	+	+	−	−	−	−	−	−	NA	NA	Death (68 days)
3a	Lammens et al. (19)	Male	16	+	NS	+	NS	NS	NS	NS	NS	NA	NA	NS
3b		2 m 3F	INA	NS	NS	+	+	NS	+	NS	NS	NA	NA	NS
3c		Male	28	+	NS	+	+	NS	+	+	NS	NA	NA	Death (24 h)
4	Vardon et al. (7)	Male	32	+	+	+	+	+	NS	NS	NS	NA	NA	Death (5 h)
5a	Wallgren-Pettersson et al. (20)	Male	28	NS	+	NS	NS	NS	NS	NS	NS	NA	NA	Death (5 min)
5b		Female	30	NS	+	NS	NS	NS	NS	NS	NS	NA	NA	Death (19 months)
5c		Female	INA	+	NS	NS	NS	NS	NS	NS	NS	NA	NA	Death (5 months)
5d		Male	31	+	+	NS	NS	NS	NS	NS	NS	NA	NA	Death (30 min)
6	Kuwata et al. (21)	Female	36	+	+	+	NS	NS	NS	NS	NS	NA	NA	NS
7a	Lawlor et al. (22)	Male	31	+	+	NS	NS	NS	NS	NS	NS	NA	NA	Death (28 days)
7b		Male	INA	+	NS	+	NS	NS	NS	NS	NS	NA	NA	Death (1 day)
8a	Yonath et al. (23)	Female	36	+	+	NS	NS	NS	NS	NS	NS	NA	NA	TOP
8b		Female	23	+	+	NS	NS	+	NS	NS	NS	NA	NA	NA
8c		Unknown	30	+	+	+	NS	NS	NS	NS	NS	NA	NA	Death (5 min)
8d		Female	20	+	+	NS	NS	+	NS	NS	NS	NA	NA	Death (2 days)
9a	Ahmed et al. (24)	Female	19	NS	NS	+	+	+	+	NS	NS	NA	NA	TOP
9b		Male	20	+	NS	+	+	NS	NS	NS	NS	NA	NA	Intrauterine Fetal demise at 27 weeks
9c		Male	21	NS	+	+	NS	+	NS	NS	NS	NA	NA	Death (8 min)
9d		Female	20	+	+	+	+	NS	NS	NS	NS	NA	NA	Intrauterine Fetal demise at 27 weeks
10	Berkenstadt et al. (12)	Female	26	+	+	+	NS	+	NS	NS	NS	NA	NA	TOP
11	Abbott et al. (25)	Female	31	NS	+	NS	+	NS	NS	NS	NS	NA	NA	Death (9 days)
12	Wang et al. (26)	Male	29	NS	+	NS	+	NS	NS	NS	NS	NA	NA	Death (2 days)
13	Avasthi et al. (10)	Unknown	INA	+	+	NS	+	NS	+	NS	NS	NA	NA	NS
14a	Yeung et al. (27)	Female	36	NS	+	NS	NS	NS	NS	NS	NS	NA	NA	Death (7 months)
14b		Male	32	+	+	NS	NS	NS	NS	NS	NS	NA	NA	Death (16 months)
14c		Female	INA	+	NS	NS	NS	+	NS	NS	NS	NA	NA	Death (49 days)
14d		Female	20	+	NS	+	NS	+	NS	NS	NS	NA	NA	TOP
15	Rocha et al. (28)	Male	13	+	NS	+	+	NS	+	NS	NS	NA	NA	TOP
16a	Zhang et al. (29)	Female	16	+	NS	+	NS	NS	NS	NS	NS	NA	NA	TOP
16b		Male	12	+	+	−	−	−	−	−	−	NA	NA	Death (3 months)
This study		Male	36	+	+	+	−	+	−	−	+	+	+	TOP
Total				25/34 (74%)	22/34 (65%)	16/34 (47%)	11/34 (32%)	9/34 (26%)	5/34 (15%)	2/34 (6%)	1/34 (3%)	1/34 (3%)	1/34 (3%)	

*GA, Gestational age; DFM, Decreased fetal movement; Poly, Polyhydramnios; Arth, Arthrogryposis; FA, Facial abnormalities; LH, Lung hypoplasia; MEE, Muscle echo enhancement; MTT, Muscle thickness thinning; TOP, Termination of pregnancy; INA, Information not applicable; NS, Not specified; NA, Not assessed.*

NM needs to be differentiated from other neuromuscular disease, such as congenital muscular dystrophy (13). It can also show decreased fetal movement, polyhydramnios, arthrogryposis on prenatal ultrasound. To the best of our knowledge, whether the enhanced echo intensity and decreased thickness of skeletal muscle also appear has not been reported in the literature. In the future, we will investigate whether other congenital myopathies also demonstrate this abnormal ultrasound performance. Congenital muscular dystrophy is associated with muscle enzymes significantly increased over 10 times, and some have white matter lesions (14). In addition, polymyositis, dermatomyositis, and mitochondrial encephalopathy also need to be distinguished from NM. Polymyositis is rare in the neonatal period. Dermatomyositis often has obvious skin lesions (15). Mitochondrial encephalomyopathy is a multisystem disease mainly involving the brain and muscle (16). Diagnosis can be further confirmed by histopathology. Congenital muscular dystrophy also show histological loss of muscle fibers and the replacement of muscle tissue by connective and adipose tissue (13). However, typical nemaline rods are characteristic of NM. Staining with Gomori trichrome or electron microscopy can substantiate the nemaline rods (17). Ultimately, WES provides a precise genetic diagnosis.

One limitation of our study was that electron microscopy was not performed because fresh specimens were not available. Another limitation is the small number of cases. We will evaluate more NM cases in the future to study the correlation between ultrasonography features and unfavorable outcomes.

## Conclusion

The large size and complexity of the genome necessitates a costly and lengthy determination of presence of neuromuscular disorders. Therefore, early abnormal ultrasound findings (decreased fetal movements, polyhydramnios, enhanced echo intensity, and decreased muscle thickness) may prompt clinicians to suspect congenital neuromuscular disease. It may provide early clue of targeted genes to facilitate a prenatal diagnosis of NM, followed by WES and biopsy to confirm the diagnosis. This is important to offer parents informed choices regarding the subsequent course of the pregnancy and to alert physicians to plan appropriate investigations. Furthermore, NM can be associated with CHD. To the best of our knowledge, this is the first report of NM associated with complex CHD.

## Data Availability Statement

The original contributions presented in this study are included in the article/Supplementary Material, further inquiries can be directed to the corresponding author.

## Ethics Statement

The studies involving human participants were reviewed and approved by Medical Ethics Committee, the Second Xiangya Hospital, Central South University. The patients/participants provided their written informed consent to participate in this study.

## Author Contributions

DL and GX contributed to conception and design of the study. DL completed the data collection and wrote the first draft of the manuscript. DL, JY, XW, YY, LY, SZ, and MZ wrote sections of the manuscript. All authors contributed to manuscript revision and read and approved the submitted version.

## Conflict of Interest

The authors declare that the research was conducted in the absence of any commercial or financial relationships that could be construed as a potential conflict of interest.

## Publisher’s Note

All claims expressed in this article are solely those of the authors and do not necessarily represent those of their affiliated organizations, or those of the publisher, the editors and the reviewers. Any product that may be evaluated in this article, or claim that may be made by its manufacturer, is not guaranteed or endorsed by the publisher.

## Supplementary Material

Supplementary Figure 1 Variant identification by Sanger sequencing. The red arrow represented the variant site (c.1516A > C).
