# Supplementary figures and images for: Case Report: Prenatal Diagnosis of Nemaline Myopathy
Source: Front Pediatr. 2022 Jul 19;10:937668. doi: 10.3389/fped.2022.937668 (PMC9343628; doi:10.3389/fped.2022.937668)

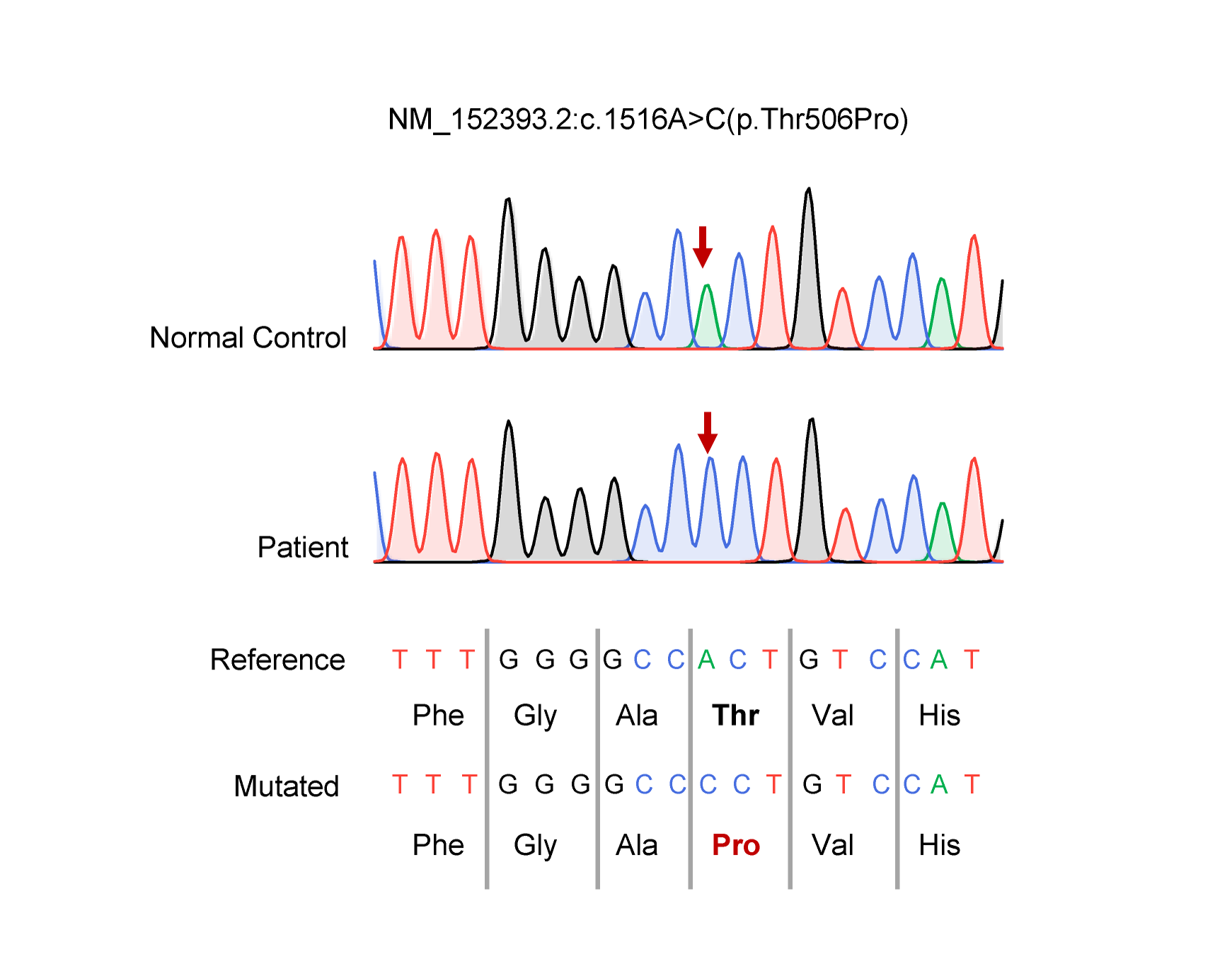

Supplement: Supplementary Figure 1 — Variant identification by Sanger sequencing. The red arrow represented the variant site (c.1516A > C). [file Image_1.TIF]
